# Mg-ZIF nanozymes disrupt the level of ROS for osteosarcoma killing via POD activity

**DOI:** 10.3389/fphar.2024.1407989

**Published:** 2024-05-06

**Authors:** Junjie Zheng, Shiqiang Zhuo, Lin Huang, Jinying Wang, Gaofeng Huang

**Affiliations:** Department of Orthopedics, Shanghai Sixth People’s Hospital Fujian, Luoshan Section, Quanzhou, China

**Keywords:** mg-ZIF, nanozymes, POD activity, Fenton reaction, ROS, osteosarcoma

## Abstract

Osteosarcoma (OS) is notorious for its high malignancy, and conventional chemotherapy drugs, while killing tumor cells, often inflict significant harm on the patient’s body. The tumor microenvironment of OS is characterized by high levels of hydrogen peroxide (H_2_O_2_). Leveraging this feature, we have developed Mg-ZIF nanoparticles, which incorporate magnesium (Mg) to confer robust peroxidase (POD)-like enzymatic activity. These Mg-ZIF nanozymes can generate highly lethal superoxide anions within tumor cells in a responsive manner, thereby achieving effective tumor destruction. Both *in vitro* and *in situ* OS models have corroborated the anti-tumor efficacy of Mg-ZIF nanozymes, while also validating their biosafety. The design of Mg-ZIF nanozymes opens a new avenue for the treatment of OS, offering a promising therapeutic strategy.

## Introduction

Osteosarcoma (OS) with a 3 per million in the general population. The occurrence of this disease is heightened during adolescence, with a particular predilection for males (Meltzer and Helman, 2021). OS typically develops in the metaphyseal growth plates of long bones, most commonly affecting the femur, tibia, and humerus (Kansara et al., 2014). The principal treatment for OS involves surgical resection. Nevertheless, solely performing a local excision is insufficient (Link et al., 1986; Bernthal et al., 2012). Without the integration of chemotherapy, patients are at a high risk of succumbing to pulmonary metastases (Kansara, Teng, Smyth and Thomas, 2014). Despite the widespread acceptance of chemotherapy in the treatment of OS, it is important not to overlook the significant negative effects it can cause, including nausea, baldness, and potential damage to the liver or kidneys (Oun et al., 2018). This motivates the ongoing search for safer and less harmful treatment options for OS.

The high levels of hydrogen peroxide (H_2_O_2_) concentration are widely recognized. This phenomenon arises from the altered metabolic processes characteristic of cancer cells (Wang et al., 2023b). Hydrogen peroxide has the ability to influence different traditional routes, which in turn control important elements of oxidative stress like cell growth, self-degradation, cell death, and resistance to medication. This ultimately supports the advancement and advancement of oxidative stress. The increased H_2_O_2_ in tumor cells can easily spread to the surrounding tumor environment, ultimately boosting the survival of OS (Zhou et al., 2015; Li et al., 2021). Therefore, a feasible strategy to combat cancer involves regulating the levels of H_2_O_2_ in OS to both kill cancer cells. Peroxidase (POD) enzyme-active substances make this strategy possible. POD-active substances catalyze the conversion of high concentrations of H_2_O_2_ within the tumor into the more cytotoxic hydroxyl radical (·OH) (Zeng et al., 2022; Wang et al., 2023a; Lu et al., 2023; Yang et al., 2023; Wei et al., 2024). The rise in reactive oxygen species (ROS) can efficiently eliminate cancer cells and has a beneficial impact on reducing the buildup of H_2_O_2_ in the tumor environment. In recent years, advances in nanomedicine have opened up new strategies for the treatment of osteosarcoma. Nanozymes, a type of nanomaterials, exhibit catalytic capabilities comparable to those of natural enzymes. They offer benefits such as affordability, convenient production on a large scale, excellent stability, and long-term preservation.

ZIFs, which are metal-organic frameworks, are created as topological isomers by combining zinc ions and imidazolate linkers (Wang et al., 2020). ZIF-derived nanoparticles have shown remarkable biocompatibility in prior studies (Mi et al., 2021). Consequently, the development of ZIFs with POD-like enzymatic activity holds promise for catalyzing Fenton reactions (Chu et al., 2023). Nanoparticles doped with magnesium (Mg) elements have been proven to possess catalytic activity for Fenton reactions; thus, doping ZIFs with Mg ions could potentially generate metal-organic frameworks with Fenton reaction catalysis capabilities. However, effectively incorporating Mg ions into the ZIF structure remains a challenge (Ge et al., 2022; Ding et al., 2023).

**SCHEME 1:**
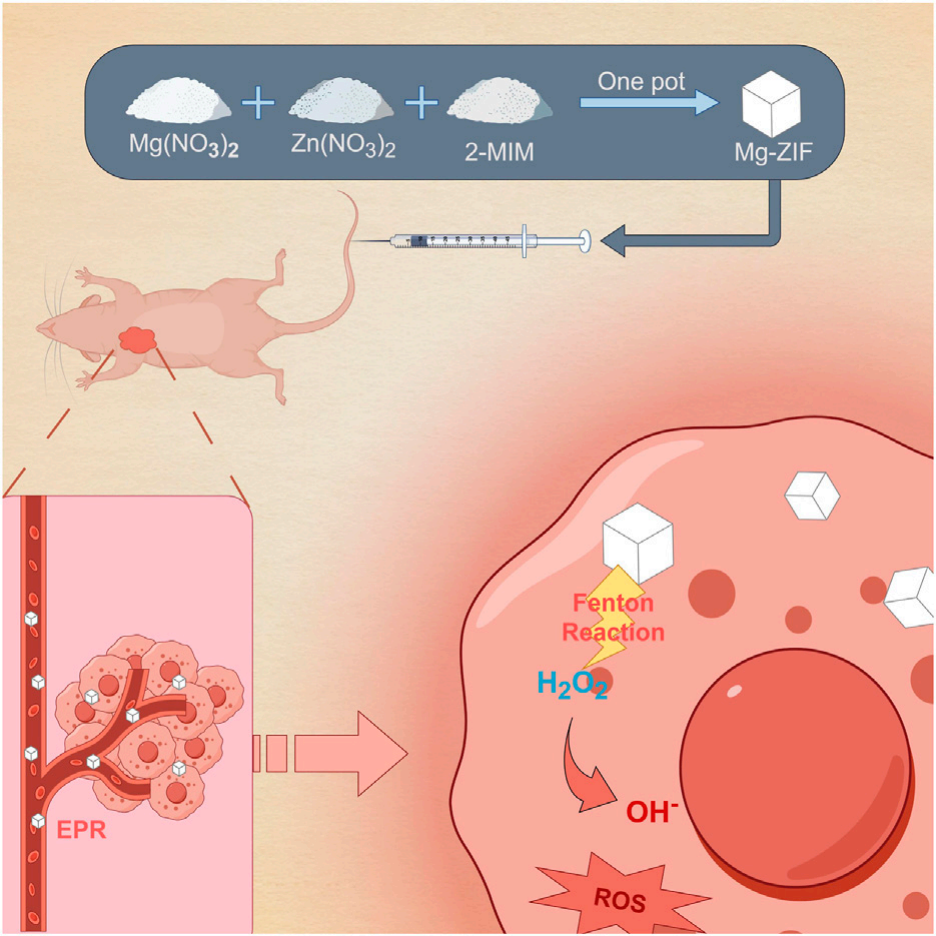
Mg-ZIF for anti-OS.

The objective of this research is to create a new ZIF that exhibits enzymatic activity similar to POD through the addition of Mg metal elements. The successfully prepared Mg-doped metal-organic framework (Mg-ZIF) show excellent POD-like enzymatic activity in the presence of H_2_O_2_. Additionally, it shows a good capability of being taken up by cells. In laboratory tests, it has been verified that Mg-ZIF is capable of producing ROS specifically at the location of the tumor, leading to the destruction of tumor cells. We further validated these findings in an *in vivo* human OS model. Moreover, this therapy exhibits good biosafety. The study introduces a fresh approach for incorporating ZIFs into cancer therapy (Scheme 1).

## Materials and methods

The methodological details provided in the Supplementary Material.

### Synthesis and characterization of Mg-ZIF

Zinc nitrate hexahydrate (4 mmol, 1.2 g) was completely dissolved in DMF (20 mL), then a solution of dimethylimidazole (12 mmol, 1.0 g) in 10 mL DMF was added slowly. After 24 h, mixture was centrifuged (10,000 rpm, 10 min) to collect precipitation. The ZIF nanoparticles were precipitated by washing with alcohol 3 times and dried.

Magnesium nitrate hexahydrate (2.0 mmol, 0.5 g), zinc nitrate hexahydrate (0.001 mol, 0.29749 g) and DMF were mixed for 0.5 h to form Mg-ZIF. Afterward, a solution of dimethylimidazole (0.006 mol, 0.4926 g) in 10 mL of DMF was added to mixture. When stirring for 24 h, mixture was centrifuged to collect precipitate. Then precipitation was washed 3 times with alcohol and dried to yield Mg-ZIF nanoparticles.

### Construction of nude mouse OS model

Female BALB/c nude mice (6W) were acquired from Beijing Vital River Laboratory Animal Technology Co., Ltd. The Animal Protection and Use Ethics Committee of Fujian Hospital affiliated with the Sixth People’s Hospital of Shanghai reviewed and approved all animal experiments (NO 2024-0007). The mice were anesthetized with pentobarbital, and a 6 mm subcutaneous injection at the chest site was administered, slowly infusing a 200 µL (2 × 10^6^ cells) suspension of 143B cells.

### Mice treatment

12 mice with tumor volumes of 100 mm^3^ were chosen and divided into three groups randomly: Control, ZIF, and Mg-ZIF, each containing four mice. The therapy was given through injection into the tail vein every 4 days, with a total of four doses. Dosages administered were as follows: the control group was given 200 µL of saline solution; the ZIF group was administered ZIF (200 mg/kg). The Mg-ZIF group was treated with Mg-ZIF (200 mg/kg). The experiment concluded on the 14 D. Throughout the therapy duration, the size of the tumor region was calculated every second day utilizing formula V = (4/3) πa × b˄2. Following the completion of the assay, the mice were euthanized. Organs from mice in every group were gathered and preserved in 4% paraformaldehyde for future slicing. The tumor areas were also excised and weighed.

## Results and discussion

### Preparation and characterization

**FIGURE 1:**
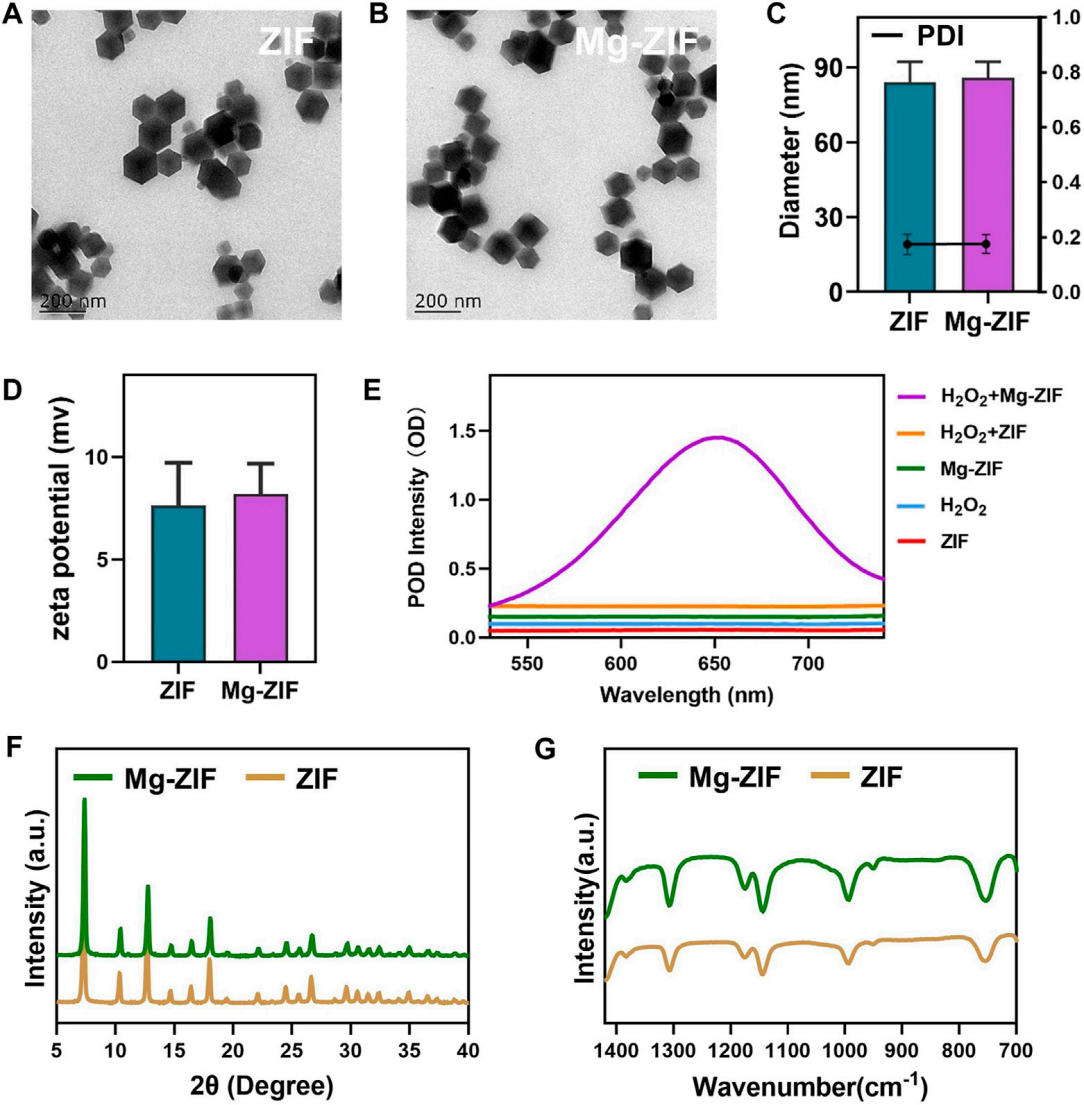
Synthesis and characteristics. **(A)** TEM of ZIF and **(B)** Mg-ZIF. **(C)** Particle dimensions and polydispersity index of produced nanozyme. **(D)** Zeta potential values for ZIF and Mg-ZIF. **(E)** ZIF or Mg-ZIF exhibit activity like that of a POD. **(F, G)** XRD and FTIR patterns of ZIF and Mg-ZIF were analyzed.

In present work, the synthesized ZIFs with enzymatic activity by altering the composition of metal ions using a one-pot method (Ye et al., 2022). The prepared ZIF and Mg-ZIF exhibited distinct morphologies (Figures 1A,B). Malvern analysis showed that the typical hydrodynamic sizes of ZIF and Mg-ZIF were 83.9 ± 5.8 nm and 85.8 ± 4.3 nm, respectively, facilitating material accumulation at the tumor location via the EPR effect. It found that the mean potentials of ZIF and Mg-ZIF were 7.6 mV and 8.2 mV, respectively, as illustrated in Figure 1D, laying the foundation for favorable cellular absorption properties. The accumulation of ROS can rapidly kill nearby tumor cells, with the higher levels of H_2_O_2_ in the tumor laying the foundation for ROS production. Following this, we examined the POD-like enzyme function of ZIF and Mg-ZIF (Figure 1E), which showed that Mg-ZIF is capable of engaging in a redox process with H_2_O_2_ to generate the more harmful ·OH.

In order to confirm the shapes of their crystals, we analyzed ZIF and Mg-ZIF through XRD and FTIR. The data showed that Mg-ZIF exhibited characteristic peaks consistent with traditionally prepared ZIF-8 (Figures 1F,G), with no impurity peaks present. The evidence presented demonstrated the high quality crystallinity of Mg-ZIF and showed that the addition of Mg metal did not impact the integrity of the ZIF crystal lattice. The data collectively demonstrate that the prepared Mg-ZIF exhibits good POD-like enzymatic activity, as well as favorable particle size and potential.

### Cytotoxicity and cellular uptake experiments

**FIGURE 2:**
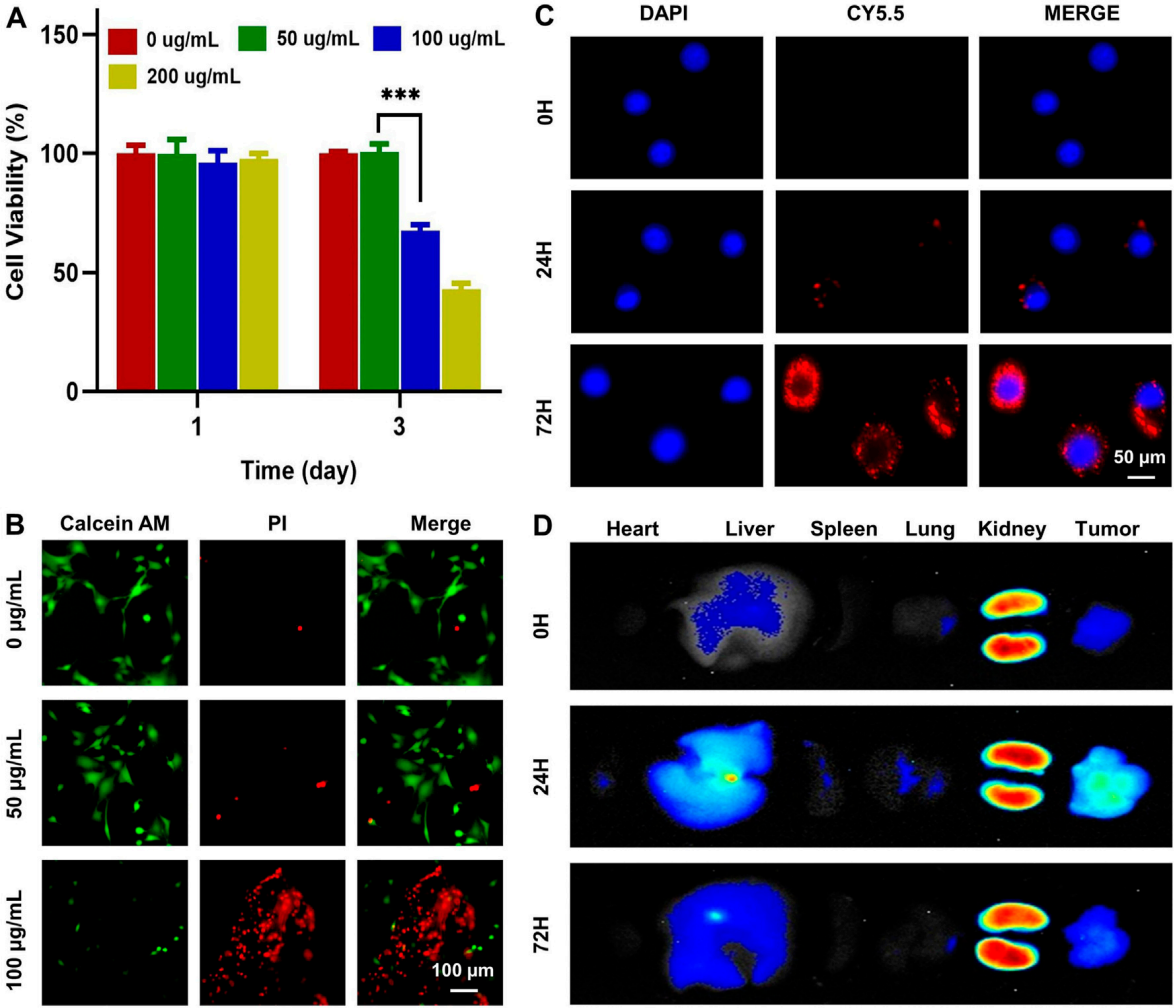
Toxicity and biodistribution of Mg-ZIF nanozymes. **(A)** CCK-8 test was used to assess the impact of Mg-ZIF nanozymes on 143B cells; **(B)** Live/dead staining was performed to demonstrate the harm caused by Mg-ZIF nanozymes on 143B cells after 72 h; **(C)** Images showing the uptake by cells were captured; **(D)** Images displaying the distribution *in vivo* were also obtained.

Cytotoxicity of Mg-ZIF nanomaterials to 143B was observed by the CCK-8 assay and live/dead assay. Dates showed that tumor cell death significantly increased after 3 days of co-culture when the Mg-ZIF concentration surpassed 100 μg/mL, as demonstrated in Figure 2A. Live/dead staining also confirmed that 100 μg/mL Mg-ZIF significantly caused cell death in 143B cells after 3 days (Figure 2B). For anti-tumor activity to occur, tumor cells must absorb Mg-ZIF. Mg-ZIF nanozymes labeled with CY5.5 (Mg-ZIF-CY5.5) were used to observe the cellular uptake process after co-culture with OS cells. The data showed that compared to 0 h, the accumulation of red fluorescence within the cells increased over time after 1 day of co-culture with Mg-ZIF-CY5.5, indicating that Mg-ZIF-CY5.5 was gradually taken up by 143B cells (Figure 2C). After a 3-day co-culture period, there was a noticeable increase in red fluorescence within 143B cells, which was due to the ongoing absorption of small nanoparticles by the cells over an extended period of time (Figure 2C).

### *In Vivo* distribution and metabolism assessment

Validating the effectiveness and compatibility of Mg-ZIF nanozymes against OS requires a thorough examination of their biodistribution in living organisms. The distribution of Mg-ZIF-CY5.5 in the body was observed at different time points following intravenous injection. A robust fluorescence signal was observed in the tumors treated with Mg-ZIF-CY5.5 1 day post intravenous administration, thanks to the improved permeability and retention effect (Figure 2D). At the same time, intense fluorescence signals were detected in the liver and kidneys, suggesting that the nanozymes were mainly taken up and processed by these organs. As time progressed (to 3 days), Mg-ZIF-CY5.5 accumulated more in the tumors.

### Anti-OS efficacy

**FIGURE 3:**
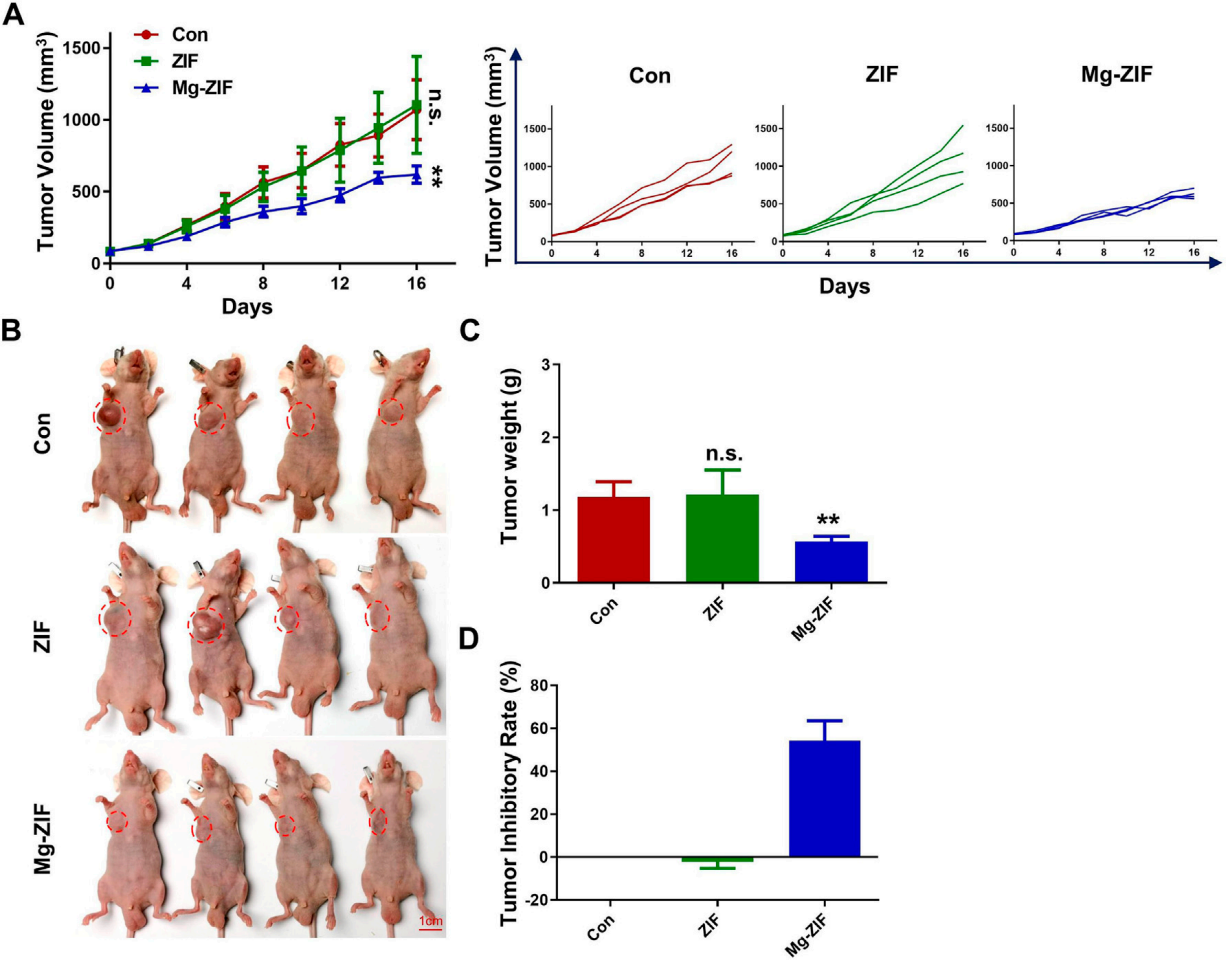
The Mg-ZIF nanozyme demonstrates *in vivo* efficacy against tumors and protects bone health. **(A)** Tumor volume advancement in all mice and individual groups. **(B)** Images of tumors. **(C)** Tumors weight. **(D)** Tumor inhibition rates.

**FIGURE 4:**
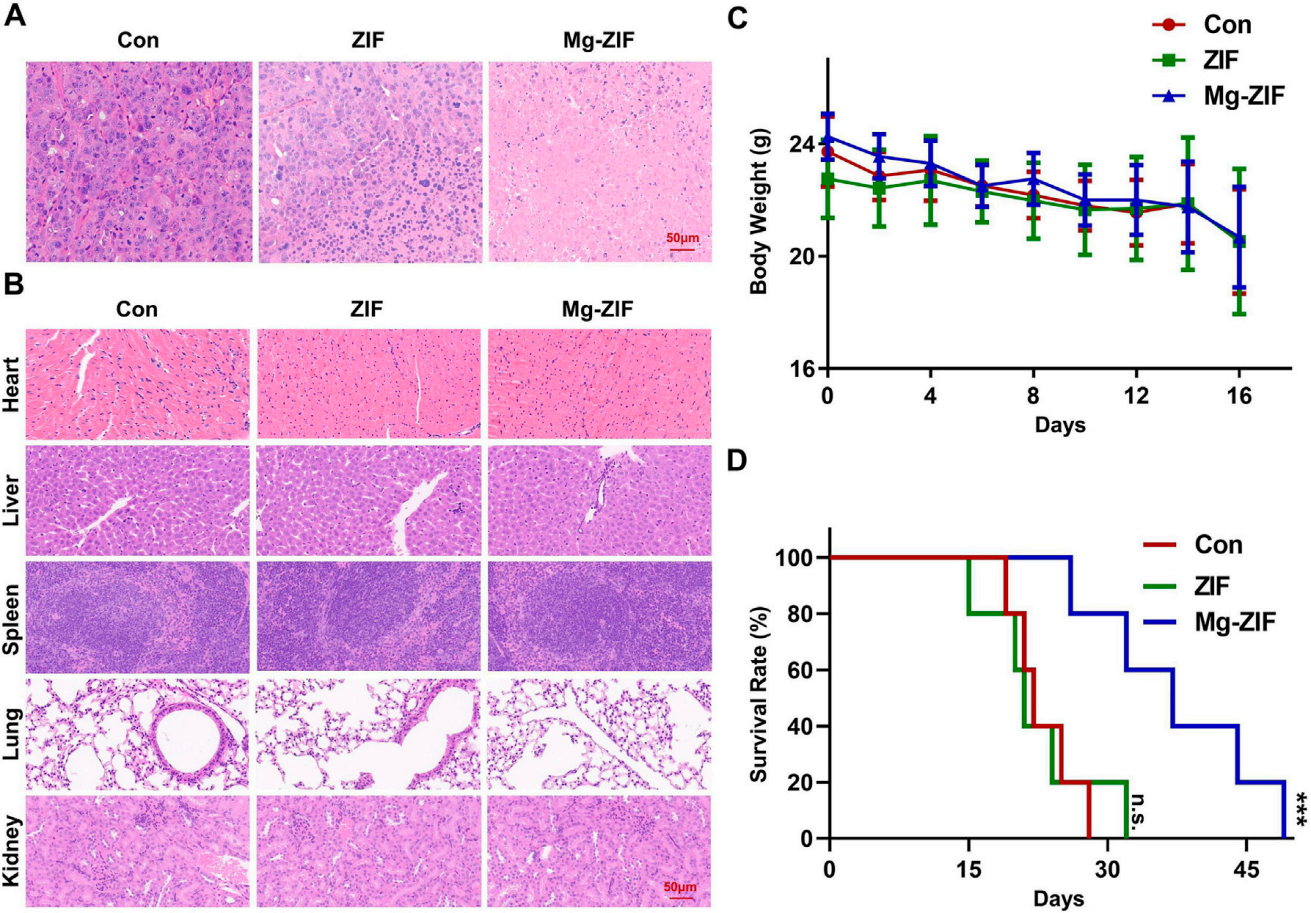
*In vivo* safety assessment. **(A, B)** Images of tumors and organs stained with hematoxylin and eosin following various treatments. **(C)** Monitoring the weight of mice following various treatments. **(D)** Analysis of mouse survival following various treatments.

The OS model in mice was established to evaluate anti-OS effects of Mg-ZIF. The Mg-ZIF group had the lowest tumor volume compared to other treatment groups. This is attributed to the Fenton reaction mediated by Mg-ZIF, which produces the more toxic ·OH, effectively killing OS cells (Figure 3A). Concurrently, in the individual tumor growth inhibition curves, no significant outliers were observed (Figure 3A). Photographs taken after the treatment showed that the tumor volume in the Mg-ZIF group was notably reduced compared to the other groups, aligning with the tumor growth inhibition curve findings. Tumor weight measurements indicated that the average tumor weight in the control groups was notably higher compared to tumors treated with Mg-ZIF (Figure 3C). Analysis of the tumor growth suppression rate showed no notable distinction between the tumors treated with ZIF nanozymes and the control group, but a marked anti-cancer impact was evident following administration of Mg-ZIF nanozymes (Figure 3D). This supports the notion that Mg-ZIF exhibits enhanced anti-tumor effects through the Fenton reaction. HE staining performed later showed that the tumor cell nuclei in the control and ZIF groups were undamaged and did not exhibit significant apoptosis, while the Mg-ZIF nanozyme-treated group displayed widespread cell death (Figure 4A). This further provides evidence for the effectiveness of Mg-ZIF nanozymes in combating OS.

### *In Vivo* safety and survival rate assessment

To evaluate the biosafety of Mg-ZIF nanozymes (Pugazhendhi et al., 2018; Songbo et al., 2019), pathological analysis was conducted on the excised major organs of mice from each treatment group (Figure 4B). HE staining results revealed no notable cellular necrosis, fragmentation, or deformation in the main organs (heart, liver, spleen, lungs, and kidneys) across all groups, demonstrating the exceptional safety and compatibility of Mg-ZIF nanozymes.

Furthermore, examination of the mice’s weight showed no notable reduction following the administration of Mg-ZIF nanozymes, indicating that the treatment did not cause harm to the mice.

Meanwhile, survival rate curves indicated that mice treated with Mg-ZIF nanozymes had longer lifespans. Compared to other groups, the survival time of tumor-bearing mice treated with Mg-ZIF nanozymes was extended by approximately 1.5 times. The data indicates that Mg-ZIF nanozymes can both successfully suppress tumor development and enhance the survival rate of mice.

## Conclusion

In summary, we developed anti-ros therapeutic nanoparticles with enzyme-like activity by combining Mg metal ions with ZIF framework (Mg-ZIF). It was found that Mg-ZIF acquired a strong ros-generating ability through POD enzyme activity. Their application in a subcutaneous OS model further confirmed the multiple effects of tumor growth inhibition and prolongation of survival through POD-like enzyme activity. Further exploration of the role of Mg-ZIF in in situ OS models is warranted in future work. More importantly, our study provides a promising strategy for OS treatment and a new vector for subsequent OS nanoparticle therapy.

## Data Availability

The original contributions presented in the study are included in the article/Supplementary Material, further inquiries can be directed to the corresponding author.
