# Supplementary material for: Mg-ZIF nanozymes disrupt the level of ROS for osteosarcoma killing via POD activity
Source: Front Pharmacol. 2024 May 6;15:1407989. doi: 10.3389/fphar.2024.1407989 (PMC11102994; doi:10.3389/fphar.2024.1407989)
Supplement: Supplementary file 1 [file DataSheet1.pdf]

Mg-ZIF nanozymes disrupt the level of ROS for osteosarcoma killing *via* POD activity

Junjie Zheng [1], Shiqiang Zhuo[1], Lin Huang [1], Jinying Wang [1] and Gaofeng Huang [1]*

Materials and Methods

Materials, Cells, and Animals

Dulbecco's Modified Eagle Medium (DMEM) was purchased from Thermo Scientific. Fetal bovine serum was obtained from Sijiqing Company. The 143B mouse osteosarcoma cells were acquired from the Cell Bank of Shanghai Institute of Life Sciences, Chinese Academy of Sciences, and cultured in high-glucose DMEM supplemented with 10% FBS, 100 μg/mL streptomycin, and 100 U/mL penicillin. The 143B cells were maintained at 37°C in a humidified atmosphere containing 5% CO2.

Cell Uptake

Mg-ZIF was mixed with an appropriate amount of FITC to obtain Mg-ZIF-FITC. Mg-ZIF-FITC was co-cultured with 143B cells for 0, 24, and 72 hours. The cells were then washed with PBS, fixed with 4% paraformaldehyde, and observed under an inverted fluorescent microscope to assess cell uptake.

In vitro Cytotoxicity Assay

The cytotoxicity of Mg-ZIF nanozymes was evaluated using a CCK-8 assay. After 143B cells adhered to the culture plates, media containing different concentrations of Mg-ZIF were added and incubated for 1 and 3 days. Subsequently, CCK-8 was added and incubated for 2 hours. Absorbance was measured at a wavelength of 450 nm using a microplate reader.

Live/Dead Staining

The interaction between 200 μg/mL Mg-ZIF nanozymes and cells was observed using the live/dead assay. After 143B cells adhered to the plates, the culture medium was removed, cells were washed with PBS, and then incubated with 2 μM Calcein-AM and 8 μM propidium iodide (PI) for 1 hour. After washing the cells with PBS, they were observed under an inverted fluorescent microscope.

Survival Analysis

Fifteen mice with tumor volumes reaching 100 mm^3 in the local tumor area were randomly

divided into Control, ZIF, and Mg-ZIF groups, with 5 mice in each group. The treatment protocol remained the same for all groups. The mice's survival status was observed for survival analysis.

Distribution and Metabolism of Materials

Mice were intravenously injected with FITC-loaded Mg-ZIF at a dose of 200 mg/kg and a volume of 200 μL through the tail vein. Mice were euthanized at 0, 24, and 72 hours post-injection to collect heart, liver, spleen, lung, and kidney for fluorescence imaging. Each mouse's various organs were photographed using a small animal live imaging system (INDEC BioSystems, USA).

H&E Staining and Imaging

Tumor tissues from mice were fixed, and previously collected formalin-fixed heart, liver, spleen, lung, and kidney samples were embedded, sectioned, and stained with H&E dye for tumor and organ histological analysis. Tumor tissues and organ tissues were observed under a microscope (Nikon Eclipse Ti, Ardmore, PA) at 20x magnification.

Statistical Analysis

Statistical analysis was performed using SPSS 18.0 software (IBM, USA) with data analyzed using Student’s T-test. A P-value < 0.05 was considered statistically significant (at a 95% confidence level). Data are presented as mean ± standard deviation (SD).
